# Interaction between Gender and Skill on Competitive State Anxiety Using the Time-to-Event Paradigm: What Roles Do Intensity, Direction, and Frequency Dimensions Play?

**DOI:** 10.3389/fpsyg.2017.00692

**Published:** 2017-05-15

**Authors:** John E. Hagan, Dietmar Pollmann, Thomas Schack

**Affiliations:** ^1^Neurocognition and Action – Biomechanics Research Group, Faculty of Psychology and Sport Sciences, Bielefeld UniversityBielefeld, Germany; ^2^Center of Excellence “Cognitive Interaction Technology”Bielefeld, Germany

**Keywords:** anxiety, intensity, interpretation, frequency, psychological skills

## Abstract

**Background and purpose:** The functional understanding and examination of competitive anxiety responses as temporal events that unfold as time-to-competition moves closer has emerged as a topical research area within the domains of sport psychology. However, little is known from an inclusive and interaction oriented perspective. Using the multidimensional anxiety theory as a framework, the present study examined the temporal patterning of competitive anxiety, focusing on the dimensions of intensity, direction, and frequency of intrusions in athletes across gender and skill level.

**Methods:** Elite and semi-elite table tennis athletes from the Ghanaian league (*N* = 90) completed a modified version of Competitive State Anxiety Inventory-2 (CSAI-2) with the inclusion of the directional and frequency of intrusion scales at three temporal phases (7 days, 2 days, and 1 h) prior to a competitive fixture.

**Results:** Multivariate Analyses of Variance repeated measures with follow-up analyses revealed significant interactions for between-subjects factors on all anxiety dimensions (intensity, direction, and frequency). Notably, elite (international) female athletes were less cognitively anxious, showed more facilitative interpretation toward somatic anxiety symptoms and experienced less frequency of somatic anxiety symptoms than their male counterparts. However, both elite groups displayed appreciable level of self-confidence. For time-to-event effects, both cognitive and somatic anxiety intensity fluctuated whereas self-confidence showed a steady rise as competition neared. Somatic anxiety debilitative interpretation slightly improved 1 h before competition whereas cognitive anxiety frequencies also increased progressively during the entire preparatory phase.

**Conclusion:** Findings suggest a more dynamic image of elite athletes’ pre-competitive anxiety responses than suggested by former studies, potentially influenced by cultural differences. The use of psychological skills interventions that require effective structure, content, and timing in a composite manner is suggested.

## Introduction

Research has consistently shown that sporting experience is often characterized by many unpleasant (negative) emotional experiences like anxiety before and during competition (Hanin, 2000a, 2007; Lazarus, 2000; Jones, 2003; Pensgaard and Duda, 2003; Skinner and Brewer, 2004; Sève et al., 2007). These negative emotions could play a significant role in performance variability, by impairing athletes’ performance (Hanin, 2000b, 2007; Vallerand and Blanchard, 2000; Jones, 2003; Pensgaard and Duda, 2003).

Anxiety is seen as an embodied process that unfolds over time and the emotional responses to it are usually characterized by affect variations due to the ever fluctuating environment athletes’ face (Lazarus, 1999; Cerin et al., 2000, 2001). Therefore, understanding the multidimensionality of athletes’ pre-competitive symptoms using the time-to-event paradigm is crucial for applied practitioners helping with preparations as competition approached (Cerin et al., 2000; Hanton et al., 2002). Although some researchers have tried examining intensity of anxiety responses in time leading up to competition (Martens et al., 1990; Jones et al., 1991; Hall et al., 1998), limited research attention has been given to the comprehensive assessment of how different dimensions of the same affect response unfold over time. Wiggins (1998) pioneered this approached through a 24 h pre-competition period (24 h, 2 h, 1 h). Other studies have subsequently been conducted using this time-to-event paradigm using different study population (Cerin et al., 2000, 2001; Woodman and Hardy, 2001; Hanton et al., 2004b; Thomas et al., 2004).

Consistently, most of these research are based on the multidimensional anxiety theory (MAT) as an approach to the examination of athletes’ pre-event, during and post competition emotional responses using the Competitive State Anxiety Inventory-2 (CSAI-2; Martens et al., 1990). The prediction of MAT indicates that if performance expectancy evaluation remains unchanged, then the intensity of both cognitive anxiety and self-confidence should remain stable in the week preceding competition. In contrast, somatic anxiety is proposed to remain stable few days prior to event but show a sudden rise and reaches its peak at the onset of competition. This is purported to dissipate once the competition begins. Though empirical support for these predictions has been forthcoming (Gould et al., 1984; Martens et al., 1990), other research findings have been contrary to the predictions (Caruso et al., 1990; Parfitt et al., 1990). Such tenuous support emphasizes the importance of providing further empirical tests of multidimensional anxiety theory. The literature base according Cerin et al. (2000) still remains equivocal. For example, researchers have noted increases or decreases in cognitive and self-confidence intensities as competition approached (Hanton et al., 2004b). A reason for these inconsistencies could rest with the lack of somewhat limited approach of examining only symptom intensity without considering additional dimensions such as direction and frequency concurrently (Jones, 1995; Woodman and Hardy, 2001).

Directional dimension is described as athletes’ interpretation of their cognitive and somatic symptom intensity as either positive or negative toward subsequent performance (Jones and Swain, 1992) while the frequency component is defined as the amount of time an athlete spent attending to the symptoms experienced concerning competition (Swain and Jones, 1993). Recent literature has supported the directional dimension with the notion that individuals can interpret the intensity of anxiety-related symptoms as either facilitative or debilitative toward upcoming performance (Mellalieu et al., 2006a).

Regarding frequency dimension, Thomas et al. (2004) asserted that emotional affect researchers have argued that individuals are more able to accurately recall and report frequency of affect than intensity of affect (Diener et al., 1991; Kardum, 1999). Again, a state where worries about competition remained at a consistent level (i.e., intensity) throughout a 1-week pre-competition period did not equate to a true cognitive state; it merely referred to the same level at two different times without providing reference to how often these symptoms are actually experienced [i.e., intensity (Swain and Jones, 1993; Jones, 1995)]. This led to the addition of a continuum to each CSAI-2 item asking “How frequently do you experience this thought or feeling at this stage?” Although intensities of anxiety components supported MAT, frequency of symptoms increased significantly through the 48 h preceding competition (48 h, 24 h, 2 h, 30 min). Frequency appears to be more sensitive to temporal changes than intensity and that states where anxiety symptoms occur for 5% of the time are considerably different from those where they are experienced 90% of the time (Swain and Jones, 1993). As a result, Thomas et al. (2004) emphasized the need for further examination of the three anxiety dimensions (intensity, direction, and frequency) using the time-to-event paradigm since results have been sparse and somewhat equivocal.

Pointing to the research of Hanton et al. (2004b) and Thomas et al. (2004), some areas were proposed for future consideration. First, if anxiety is considered as a complex set of emotions that unfold over time, then examining it using new research methods like the Experience Sampling Method (ESM) would be significant step toward eliciting a more dynamic image of athletes’ pre-competition emotional experiences (Cerin et al., 2001). In ESM, participants provide standardized descriptions of their momentary thoughts, feelings, and behaviors across a range of situations encountered in their daily training, thereby allowing researchers to sample a broad range of variables in different environmental context (Stone and Shiffman, 1994).

Significantly, measuring variables like gender and skill level that may moderate temporal responses of athletes’ emotional experiences using a high standard population over time with a more inclusive and interaction approach will aid further understanding in anxiety literature (Krane, 1995; Hanton et al., 2004b). For example, research has noted that gender moderates temporal patterning of anxiety in several studies (e.g., Jones and Cale, 1989; Donzelli et al., 1990; Jones et al., 1991; Swain and Jones, 1993). Jones and Cale (1989) found that males showed no changes on the cognitive and self-confidence sub-scales of the CSAI-2 during the pre-competition period. However, females reported a gradual elevation in scores with a simultaneous increase in the intensity of somatic symptoms and a decline in self-confidence. Similarly, an investigation into temporal responses regarding between-subject variable of skill level (elite/non-elite) revealed skill level differences restricted to the directional anxiety dimension. The elite group showed more facilitative interpretation toward cognitive and somatic symptoms than the non-elite group. Additionally, temporal changes were identified across cognitive, somatic, and self-confidence intensity and frequency with greater temporal variation observed in the frequency dimension (Hanton et al., 2004b). Other studies have shown that athletes high in skill level can experience lower intensities (Gal-Or et al., 1986; Campbell and Jones, 1997), and more facilitative interpretations (Jones et al., 1994), of the symptoms associated with competitive anxiety for time periods immediately before competition.

As to whether these two moderating variables would interact in one research design is yet to be documented. Additionally, uncharted in the increasing volume of cross-cultural research is studies exploring the influence of African culture on the affective experiences of competitive athletes of African descent. For instance, somatization literature from cultural psychology perspective suggests that in many parts of the world, the body and mind are connected in the expression of distress (Dzokoto, 2010). The base rates of this phenomenon have been found to vary both within and across cultures, with non-western cultures being associated with more reports of physical symptoms in psychologically distressed individuals (Kleinman and Good, 1985; Kirmayer et al., 1998). It would therefore be justifiable to investigate pre-competitive anxiety experiences in an African setting to understand the possible cultural influences on these aforementioned variables.

Aside the above illustrations, athletes’ affective response to competition is also thought to be dependent on task characteristics and requirements of the sport (Krane and Williams, 1987; Martens et al., 1990; Jones et al., 1991; Hassmen and Blomstrand, 1995) and that there may be more pressure and personal exposure associated with individual sports than team sports (Woodman and Hardy, 2003). A sport like table tennis places series of emotional and cognitive demands on performers due to its task characteristics and situational demands. Like other fast paced and reactive games, the short response window is often dictated by the speed of the ball which forces performers to use advanced cues to decide what response is required and how that movement should be carried out (Krohne and Hindel, 1988; Krohne, 1989; Abernethy, 1991). Each distraction and emotional instability are likely to result in a series of faults, and very likely, to affect the outcome of a match.

Therefore, understanding pre-event competitive anxiety responses in the context professional practice in table tennis, not only holds practical importance for consultants and coaches working with different standards of athletes under fluctuating emotional experiences but also aid theoretical and empirical enquiry (Mellalieu et al., 2006b; Neil et al., 2006). Again, these issues could provide an empirical framework for the structure, timing, and content of temporal psychological skills interventions designed to assist performers affected by their pre-event mental states (Thomas et al., 2007).

The purpose of the present study was to draw together some of the issues from the prevailing literature. First, the study examined the relative independence of competitive state anxiety dimensions (intensity, direction, and frequency) as separate components of athletes’ emotional experience. It was hypothesized that the intensity and directional perceptions, and the intensity and frequency of intrusions of competitive anxiety would relatively be independent. Second, as a central focus of this study, it was to establish the extent to which gender and skill level interact across anxiety intensity, direction, frequency, and time-to-competition. Specifically, it was proposed that elite (international) male athletes would experience lower intensities of cognitive and somatic anxiety, and exhibit higher intensities of self-confidence than elite (international) female counterparts and that these differences would remain throughout the pre-competition period. It was further hypothesized that elite males athletes would interpret symptoms associated with cognitive and somatic anxiety as more facilitative toward upcoming performance than elite females and that these differences would remain throughout the pre-competition period. For frequency of intrusions of anxiety and self-confidence, it was hypothesized that elite male athletes would experience less frequency of intrusions for cognitive anxiety and somatic anxiety, and greater frequency of intrusions for self-confidence in comparison to the elite females athletes and that these differences would remain throughout the pre-competition period. With respect to semi-elites (national), it was predicted that there would be fluctuations in the anxiety dimensions between males and females across the pre-competition period but no opinion was offered on which group will differ. Third, MAT predictions were also assessed. It was predicted that cognitive anxiety and self-confidence intensity would remain stable in the time leading up to competition whereas somatic intensity would increase just prior to competition. For the direction dimension, no change over time effects would be found. Besides, cognitive and somatic symptoms would increase as the competition moved closer but self-confidence would remain stable for the frequency dimension.

## Materials and Methods

### Participant Selection Criteria

Purposive sampling was used to select participants who reflected issues pertaining to this research direction (Patton, 2002). The criteria for elite (international) status were that participants should have achieved national honors and represented their country in international events at different stages of their playing career, a procedure similar to one adopted by Hanton et al. (2005). Semi-elite (national) participants were those who had currently achieved district and/or regional honors and have consistently competed in the national table tennis championship/league over five competitive seasons.

### Participants

The survey procedure was approved by the Institutional Review Board (IRB) at Bielefeld University, and adhered to the ethical standards of the sixth revision of the Declaration of Helsinki. The National Sports Authority and Table Tennis Federation in Ghana were contacted to establish links with elite sporting performers who competed in the national league through their respective clubs. Athletes who fit the elite criteria were contacted, informed of the nature of the study, gave their written consent and completed the modified CSAI-2 inventory. Confidentiality and anonymity of all study participants were preserved at all stages of the investigation and that data collected remained confidential and solely for academic purposes. Participants were told that the study’s purpose was to gain an in-depth understanding of their emotional experiences prior to a competitive league fixture. The actual sample size was ninety (*N* = 90) with players ages ranged from 15 to 39 years (*M* = 26.26, *SD* = 5.29). Of these athletes, *N* = 35 (38.9%) were females whereas *N* = 55 (61.1%) were males. Furthermore, of these classification, *N* = 47 (52.2%) were noted as elite (international), with *N* = 43 (47.8%) named as semi-elite (national). Of the elite athletes, *N* = 21 (44.6%) were females compared to *N* = 26 (55.4%) for males while semi-elite athletes, *N* = 14 (32.6%) were females in contrast with *N* = 29 (67.4%) for males, all with at least 3 years of competitive playing experience (*M* = 9.63, *SD* = 5.12), trains four times a week on the average.

### Instrumentation

#### Modified CSAI-2

The modified version of the CSAI-2 was used to measure intensity and direction of pre-performance cognitive anxiety, somatic anxiety and self-confidence with nine items in each subscale. Participants rated the intensity of each item on a scale anchored by 1 (“not at all”) and 4 (“very much so”), with overall subscale intensity scores ranging from 9 to 36. Satisfactory internal consistency for the intensity of the subscales has been reported previously with Cronbach alpha coefficients ranging from 0.79 to 0.90 (Martens et al., 1990). Jones and Swain’s (1992) direction scale was included for each item in which each respondent rated the degree to which the experienced intensity of each symptom was either facilitative or debilitative to subsequent performance using a bipolar scale ranging from -3 (“very debilitative”) to +3 (“very facilitative”). Overall subscale direction scores ranged from -27 to +27, where a negative score denoted a debilitative state and a positive score as facilitative experience. Four studies have reported internal reliability coefficients for the direction subscales, ranging from 0.72 to 0.90 (Jones and Hanton, 1996; Swain and Jones, 1996; Wiggins, 1998; Hanton et al., 2000). The frequency of intrusions scale, developed by Swain and Jones (1993), was also included in the modified CSAI-2. This scale assesses the degree to which symptom related thoughts or experiences occurred on a scale ranging from 1 (‘not at all’) to 7 (‘all the time’) for each item of the inventory. Reported internal reliability coefficient of this scale ranged from 0.70 to 0.93 (Thomas et al., 2004). In the current study, reliability scores reported ranged from 0.77 to 0.80 for the intensity dimension, 0.80 to 0.82 for the direction dimension and 0.77 to 0.80, values that are consistent with previous research and considered satisfactory.

### Procedure

Using the ESM approach, pre-competitive anxiety symptoms were measured at three different stages (7 days, 2 days, 1 h) in a 7-day interval with the aid of a signal to trigger the completion of the anxiety instrument. To minimize memory disturbance through intrusions, the survey was done at a 3-week interval for the temporal period (7 days, 2 days, 1 h), spread across the period of data collection and for the same league competition. Participants were informed and paged randomly once a day during their practice sessions in the 7 days leading up to a competitive Ghana Table Tennis League (GTTL) fixture. These were repeated in the subsequent weeks in the other of assessment schedules. Prior to the assessment, an introductory session was held to brief participants on the use of the signal, explained the concepts of cognitive, somatic, and self-confidence symptoms associated to competitive anxiety, and educated on the difference between these psychological constructs. Participants were required to note their intensity, interpretation and frequency of cognitive, somatic symptoms and self-confidence experienced during the predetermined periods at the normal 7-day competitive cycle. Athletes’ responses on the modified CSAI-2 inventory were taken as soon as possible after receiving a signal.

### Data Analysis

Data analysis was carried out in different stages. First, data prescreening procedures were conducted to investigate the accuracy of the data and statistical assumptions. Second, different anxiety dimensions and self-confidence through correlational analysis to assess their relative independence at the pre-competition stage was computed. Third, pre-competition means were also calculated. Fourth, the possible influence of gender and skill level over the modified CSAI-2, self-confidence, using separate Multivariate Analyses of Variance (MANOVA) procedures testing for interaction and main effects of gender and skill by time-to-competition (repeated measures) on the three dimensions. Univariate Analyses of Variance (ANOVA) with Bonferroni adjustments for CSAI-2 and self-confidence subscales was employed for follow-up analyses to determine where the differences rested (Tabachnick and Fidell, 1996; Field, 2000). Sphericity assumption was also assessed by means of Mauchly’s test in the within-subject repeated measure analyses, and whenever the test was violated, necessary technical corrections were performed using the Greenhouse–Geisser test (Tabachnick and Fidell, 1996; Field, 2000).

## Results

### Data Pre-screening

Assumptions of univariate and multivariate analyses including missing cases and distributions were tested (Tabachnick and Fidell, 1996; Field, 2000). No missing cases and univariate or multivariate outliers were identified through Mahalanobis distance test. In addition, assumptions of normality, linearity, multicollinearity, and singularity were deemed satisfactory. However, the assumption of equality of covariance matrices, although satisfactory at the univariate level (Levene’s test and *F*_max_ ratios), was violated in some cases at the multivariate level (Box’s test). Therefore, Pillai’s trace was chosen as the multivariate test statistic due to its robustness over violation (Tabachnick and Fidell, 1996; Field, 2000).

### Correlational Analysis

Correlations between the intensity and direction scales of anxiety further strengthen the significance of separate assessment of these constructs because of their degree of independence. The coefficient values obtained between intensity and direction dimensions of cognitive and somatic anxiety showed a maximum shared variance proportion of 8% (*r* = 0.29). This result is similar to previously reported findings (Hanton et al., 2000, 2004b; Jones and Hanton, 2001; Thomas et al., 2004). However, the variance proportions displayed for the self-confidence construct revealed a level of shared variance of 26% (*r* = 0.51) between the two scales. This debunks previous directional perceptions research notion that self-confidence directional scale essentially measures the same state as the intensity scale (Jones et al., 1993; Jones and Hanton, 2001), calling for the self-confidence direction subscale to be discarded for future research based on correlation coefficient of 0.80 obtained between the two variables by Jones et al. (1993). The proportion obtained in this study further illustrates a degree of independence between the two constructs, a view echoed by Hanton et al. (2004b). With respect to the intensity and frequency dimensions, results further make a strong case for measuring these separate dimensions of emotional response although figures reported in this study were higher than those reported in other studies (Diener et al., 1991; Kardum, 1999; Hanton et al., 2004b; Thomas et al., 2004). Cognitive intensity and frequency displayed a shared variance of 38% (*r =* 0.62), with a slightly decreased shared variance of 30% (*r* = 0.55) for somatic intensity and frequency, whereas self-confidence intensity and frequency shared the least 24% (*r* = 0.49) variance during the pre-competitive phase. These findings further advances claim that both intensity and frequency dimensions should be considered as separate constructs of emotional response that warrant separate measurement although related (Diener et al., 1991; Kardum, 1999; Hanton et al., 2004b).

### Multivariate Analysis of Variance

A 2 (Gender) × 2 (skill level) × 3 (time-to-event) MANOVA, with repeated measures were computed. One MANOVA was conducted on each anxiety dimension (intensity, direction, and frequency of intrusions), with gender and skill level acting as the independent variables while cognitive anxiety, somatic anxiety, and self-confidence acted as the dependent variables over all time periods in each analysis. Interaction effects were observed for anxiety (*p* < 0.05) suggesting that any change-over-time patterns were not similar across the two between-subject factors. Subsequently, data was presented across gender and skill level for the change-over- time analysis (**Table 1**).

**Table 1:** Means and standard deviations on overall sub-scales for anxiety and self-confidence across all periods by gender and skill level.

		Male		Female	
Variable	Period	Elite *M* (*SD*)	Semi-elite *M* (*SD*)	Elite *M* (*SD*)	Semi-elite *M* (*SD*)
CA-I	7 days	25.12 (4.31)	22.38 (4.22)	19.43 (4.80)	22.43 (4.10)
	2 days	20.12 (4.31)	17.38 (4.22)	14.67 (4.52)	17.42 (4.10)
	1 h	29.12 (4.31)	26.38 (4.22)	23.43 (4.80)	26.43 (4.10)
SA-I	7 days	20.19 (3.88)	18.45 (3.71)	16.38 (4.01)	17.57 (4.22)
	2 days	17.19 (3.88)	15.45 (3.71)	13.38 (4.01)	14.57 (4.22)
	1 h	24.19 (3.88)	22.45 (3.71)	20.38 (4.01)	21.57 (4.22)
SC-I	7 days	30.58 (2.82)	28.28 (5.26)	30.52 (2.64)	30.14 (4.94)
	2 days	32.58 (2.82)	30.27 (5.26)	32.52 (2.64)	32.14 (4.94)
	1 h	34.58 (2.82)	32.27 (5.26)	34.52 (2.64)	34.14 (4.94)
CA-D	7 days	6.96 (14.05)	6.27 (13.42)	8.14 (12.41)	6.43 (14.41)
	2 days	5.19 (12.28)	4.79 (13.27)	7.28 (11.32)	4.50 (12.79)
	1 h	8.23 (13.76)	6.79 (14.98)	9.90 (12.53)	9.64 (13.18)
SA-D	7 days	-4.69 (13.49)	-2.38 (12.04)	3.14 (13.01)	-5.42 (12.16)
	2 days	-6.46 (13.28)	-3.52 (12.00)	1.71 (12.62)	-6.57 (10.78)
	1 h	-5.07 (12.94)	0.93 (13.38)	4.52 (13.26)	-3.71 (13.09)
SC-D	7 days	17.26 (3.59)	15.65 (4.88)	17.66 (4.36)	21.07 (4.10)
	2 days	14.65 (3.31)	12.79 (4.79)	14.66 (4.36)	18.21 (3.78)
	1 h	20.26 (3.59)	18.65 (4.87)	20.66 (4.36)	24.07 (4.10)
CA-F	7 days	37.34 (8.94)	32.24 (8.57)	30.04 (9.35)	32.21 (7.29)
	2 days	40.34 (8.94)	35.24 (8.57)	33.04 (9.35)	35.21 (7.29)
	1 h	42.34 (8.94)	37.24 (8.57)	35.04 (9.35)	37.21 (7.29)
SA-F	7 days	30.26 (8.62)	25.75 (6.65)	20.52 (4.78)	24.21 (8.25)
	2 days	28.26 (8.62)	23.75 (6.65)	18.52 (4.78)	22,21 (8.25)
	1 h	34.26 (8.62)	29.75 (6.65)	24.52 (4.78)	28.21 (8.25)
SC-F	7 days	47.26 (6.58)	42.96 (10.12)	47.14 (6.44)	47.64 (10.21)
	2 days	49.26 (6.58)	44.96 (10.12)	49.14 (6.44)	49.64 (10.21)
	1 h	51.26 (6.58)	46.96 (10.12)	51.14 (6.44)	51.64 (10.21)

*CA, cognitive anxiety; SA, somatic anxiety; SC, self-confidence; I; intensity; D, directional interpretation; F, frequency of intrusions.*

### Gender and Skill Level Impact

Significant between-subjects interactions were observed in the MANOVAs testing for the impact of gender and skill level on intensity, Pillai’s trace = 0.107, *F*(3,84) = 3.338, *p* < 0.05, $\eta_{p}^{2}$ = 0.10; direction, Pillai’s trace = 0.119, *F*(3,84) = 3.778, *p* < 0.05, $\eta_{p}^{2}$ = 0.11; frequency, Pillai’s trace = 0.96, *F*(3,84) = 2.971, *p* < 0.05, $\eta_{p}^{2}$ = 0.09 regardless of time period. However, across all analyses, no within-group for time-to-event significant interaction was noted (*p* > 0.05) suggesting that any change over patterns were similar across gender and skill level.

### Anxiety Intensity Dimension

**FIGURE 1:**
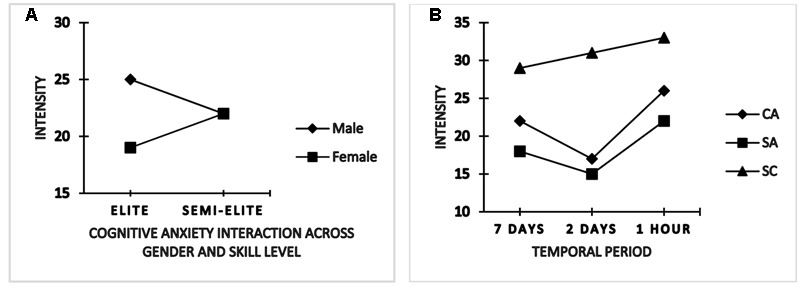
**Interaction and time-to-event anxiety and self-confidence intensity changes**.

A significant between-subjects (gender × skill level) interaction effect was noted for only cognitive state anxiety intensity, *F*(1,86) = 8.826, *p* < 0.05, $\eta_{p}^{2}$ = 0.09. A further inspection of the follow-up test revealed both inter and intra group variance. Specifically, elite (international) female athletes were less cognitively anxious (*M* = 19.17) than their elite (international) male counterparts (24.78). There was no significant difference between semi-elite (national) female and the semi-elite (national) male group as noted by their mean values (*M* = 22.09 vs. 22.04), respectively. Elite (international) females exhibited lower cognitive symptoms than their semi-elite (national) group (*M* = 19.17 vs. 22.09) whereas elite (international) males were more cognitively anxious than the semi-elite (national) male counterparts (*M* = 24.78 vs. 22.04) (**Figure 1A**).

A significant multivariate main effect was noted for gender regardless of time period, Pillai’s trace = 0.112, *F*(3,84) = 3.541, *p* < 0.05, $\eta_{p}^{2}$ = 0.11. A follow-up between-subject ANOVAs indicating gender differences were noted for cognitive state anxiety, *F*(1,86) = 8.521, *p* < 0.05, $\eta_{p}^{2}$ = 0.09 and somatic state anxiety, *F*(1,86) = 7.472, *p* < 0.05, $\eta_{p}^{2}$ = 0.08, respectively. A corrected *t*-test showed males experienced greater intensity of both cognitive and somatic anxiety symptoms than their female group (*M* = 23.14 vs. 19.65; 20.63 vs. 17.31) throughout the preparation period prior to competition (**Table 1**).

In addition, time-to-event main effect was also observed in the intensity dimension, Pillai’s trace = 0.997, *F*(1,86) = 2.923, *p* < 0.001, $\eta_{p}^{2}$ = 0.99, with a follow-up within-subject ANOVA noting changes for cognitive state anxiety, *F*(2,172) = 3.651, *p* < 0.001, $\eta_{p}^{2}$ = 0.99 and somatic state anxiety, *F*(2,172) = 2.874, *p* < 0.001, $\eta_{p}^{2}$ = 0.94, respectively. An inspection of the corrected *t*-test indicated both cognitive and somatic state anxiety intensity symptoms declined 2 days prior to competition after an initial increase 7 days before but sharply increased 1 h than the other days before, depicting a temporal fluctuation during the preparation period (**Figure 1B**).

### Anxiety Direction Dimension

**FIGURE 2:**
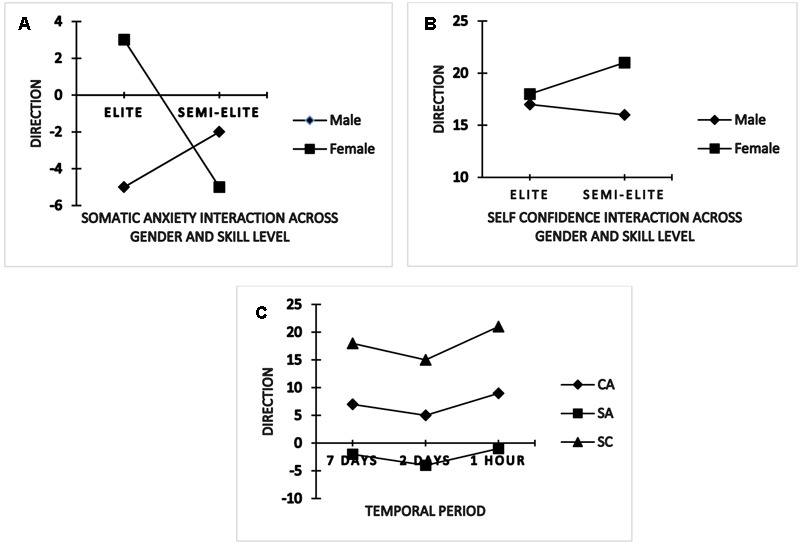
**Interactions and time-to-event anxiety and self-confidence directional changes**.

State anxiety direction findings revealed a significant between-group (gender × skill level) interaction for somatic state anxiety, *F*(1,86) = 4.377, *p* < 0.05, $\eta_{p}^{2}$ = 0.04 and self-confidence *F*(1,86) = 7.765, *p* < 0.05, $\eta_{p}^{2}$ = 0.08. A further inspection of the follow-up test revealed both inter and intra group differences. Notably, elite (international) female athletes exhibited a positive somatic anxiety response (*M* = 3.12) compared to the elite (international) male counterparts by interpreting a similar intensity in a negative manner (*M* = -5.41). However, semi-elite (national) females exhibited a more debilitative response (*M* = -5.23) compared to the semi-elite (national) male group (*M* = -2.27). In addition, elite (international) females showed facilitative interpretation to somatic anxiety symptom responses than the debilitative signs showed by the semi-elite (national) female group (*M* = 3.12 vs. -5.23) while both international and national male athletes interpreted their somatic anxiety symptoms in a negative manner (*M* = -5.41 vs. -2.27) (**Figure 2A**).

For self-confidence, both elite (international) females and males showed similar intensity response (*M* = 17.66 vs. 17.39) and subsequently perceived this to be facilitative toward their performance. A variation instead was noted for semi-elite (national) females who demonstrated more self-confidence intensity response compared to their male counterparts (*M* = 21.11 vs. 15.70). Further, international females were less self-confident than the national female group (*M* = 17.66 vs. 21.11) whereas international males were more self-confident than their national male group (*M* = 17.39 vs. 15.70) (**Figure 2B**).

A significant main effect was observed for gender regardless of time period, Pillai’s trace = 0.127, *F*(3,84) = 4.057, *p* < 0.05, $\eta_{p}^{2}$ = 0.12. A follow-up between-subject ANOVA noting gender difference for only self-confidence, *F*(1,86) = 9.475, *p* < 0.05, $\eta_{p}^{2}$ = 0.09. Specifically, female athletes observed a more facilitative interpretation of symptoms associated with self-confidence than their male counterparts (*M* = 19.39 vs. 16.54) throughout the pre-competition period (**Table 1**).

Also, time-to-event main effects were noted in the direction dimension, Pillai’s trace = 0.940, *F*(5,82) = 2.950, *p* < 0.001, $\eta_{p}^{2}$ = 0.94, with within-subject ANOVA showing changes for cognitive state anxiety, *F*(2,172) = 16.956, *p* < 0.001, $\eta_{p}^{2}$ = 0.16, somatic state anxiety, *F*(2,172) = 14.824, *p* < 0.001, $\eta_{p}^{2}$ = 0.14 and self-confidence, *F*(2,172) = 15.867, *p* < 0.001, $\eta_{p}^{2}$ = 0.94. Corrected *t*-tests further showed directional perception of cognitive state anxiety becoming more facilitative 1 h before competition whereas somatic state anxiety debilitative interpretation was more pronounced 2 days prior to the event but slightly improved 1 h before competition. Self-confidence displayed was quite high 1 h compared to the other days prior to competition (**Figure 2C**).

### Anxiety Frequency Dimension

**FIGURE 3:**
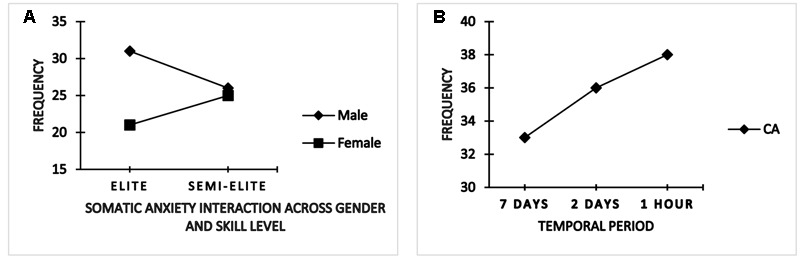
**Interaction and time-to-event changes for somatic and cognitive anxiety frequency**.

A significant between-subjects (gender × skill level) interaction effect was observed for only somatic state anxiety frequency, *F*(1,86) = 6.776, *p* < 0.05, $\eta_{p}^{2}$ = 0.07. A follow-up test revealed that elite (international) female athletes experience less frequency somatic anxiety symptoms (*M* = 21.19) in comparison to their elite (international) male counterparts (*M* = 30.93) throughout the pre-competition phase. Similar observation was noted between semi-elite females (national) versus semi-elite (national) male (*M* = 24.85; 26.42) group, respectively. Further, elite (international) females were less somatically anxious than their semi-elite (national) female group (*M* = 21.19 vs. 24.88) whereas elite (international) males were more somatically anxious than the semi-elite (national) male counterparts (*M* = 30.93 vs. 26.42) (**Figure 3A**).

A significant main effect was noted in the frequency dimension for gender, Pillai’s trace = 0.141, *F*(3,84) = 4.614, *p* < 0.05, $\eta_{p}^{2}$ = 0.14. A follow-up between-subject ANOVA observed gender difference for only somatic anxiety, *F*(1,86) = 12.841, *p* < 0.05, $\eta_{p}^{2}$ = 0.13. A corrected *t*-test suggested females experienced less frequency of somatic anxiety symptoms throughout the pre-competition period in comparison to their male group (*M* = 23.03 vs. 28.68) (**Table 1**).

Time-to-event main effect was also noted; Pillai’s trace = 0.990, *F*(1,86) = 7.041, *p* < 0.001, $\eta_{p}^{2}$ = 0.09, with a follow-up within-subject ANOVA indicating changes for only cognitive anxiety frequency, *F*(2,172) = 15.72, *p* < 0.001, $\eta_{p}^{2}$ = 0.99. A corrected *t*-test indicated frequency of cognitive symptoms increased progressively between 7 days, 2 days, and 1 h preceding competition (**Figure 3B**).

## Discussion

The major strength of this study is the examination of the much proposed dimensions of competitive anxiety (intensity, direction, and frequency: Hanton et al., 2004b; Thomas et al., 2004) from an interaction perspective that previous research has ignored within one quantitative research design. It was also in response for more inclusive sport psychology research involving high standard athletes across gender (Perry and Williams, 1998; Woodman and Hardy, 2003). From a theoretical perspective, the correlational findings emphasized the significance of assessing the anxiety dimensions separately through their level of shared variance. Obtained coefficient values between intensity, direction, and frequency gave credence to values reported in other temporal studies for times before competition (Hanton et al., 2004b; Thomas et al., 2004). The divergence between intensity, direction, and frequency of responses further strengthens these anxiety constructs as separate dimensions that independently contribute to individuals’ affective experience, although very related (Diener et al., 1991; Kardum, 1999).

Findings indicated significant interactions for between-subjects factors gender and skill level, highlighting significant differences between the elite (international) groups and the semi-elite (national) groups for intensity, direction, and frequency of intrusions. However, across all analyses, no within-group for time-to-event significant interaction was noted, suggesting that any change over patterns were similar across gender and skill level. The between-subjects interpersonal factors did influence anxiety responses, but only partly as hypothesized. Interestingly, elite (international) female athletes did not report higher intensities in cognitive anxiety symptoms as compared to their elite (international) male counterparts, a finding that is similar to results reported in other studies (Perry and Williams, 1998; Woodman and Hardy, 2003). This finding contradicts previous research on a commonly held belief that women, compared with men, mostly report higher symptoms of cognitive anxiety (Gill, 1988; Martens et al., 1990; Russell et al., 1998) and less stable before competing (Jones and Cale, 1989; Jones et al., 1991). Further, the current findings challenges the assertion made by Jones et al. (1994) and Jones and Swain (1995) that highly and intermediate skilled athletes may exhibit similar cognitive anxiety intensity symptoms because elite (international) females exhibited lower cognitive symptoms than their semi-elite (national) group whereas elite (international) males were more cognitively anxious than the semi-elite (national) male counterparts. When the between-subjects factor were considered separately, males experienced greater intensity of both cognitive and somatic anxiety symptoms than their female group regardless of skill level.

Taken collectively, and most significantly, cultural differences may have potentially influenced the above findings in this study. For example, ethnic identity is defined as a multidimensional construct which encompasses one’s sense of belonging to an ethnic group as well as shaping of thoughts, feelings, perceptions, manners, and behaviors associated with that ethnic group’s membership (concept of self: Phinney and Rotheram, 1987; Markus and Kitayama, 1991). Hence, the display of emotions cannot be considered to be universal across other societies. Anecdotal evidence suggest that males in Ghana, like others from many African societies, are overtly expected to show bravery, resilience, and some sort of hardiness toward unpleasant emotional episodes like anxiety, distress, and depression. These experiences could cause elevated levels of apprehension, panic, fear, and other physiological manifestations within individuals (Fischer, 2000). Within Ghanaian culture, males who openly display these unpleasant emotional experiences are branded as lacking competitiveness, desire to succeed, and goal orientation motives and are often confronted with shame, guilt and subsequent rejection because of their perceived cowardice attitude and/or behaviors. There seems to be lack of cross-cultural studies comparing African athletes against their Western counterparts using the CSAI-2. Future research should consider this approach since there are diverse social behaviors and internal processes by virtue of one’s cultural identity. Other plausible explanations for these findings are, first, the change in perception of women’s sport and the improvement position of women in sports as compared to the past decades, where past research was conducted, might be a possible explanation.

Additionally, the males’ event in this study was characterized by heavy playing schedules, serious competition for team places for their impending African championship, media and the fans predominant attention as compared to the females’ event could have potentially evoked elevated levels of both cognitive and somatic symptoms in the male performers (Heather, 2010). There seems to be also lack of studies involving truly elite athletes, posing a serious problem in terms of generalization of research findings to elite performers (Woodman and Hardy, 2003). Therefore, findings in competitive situations have exaggerated gender differences in cognitions, especially with females (Gill et al., 1984). For instance, the stress that elite athletes endure may be rather different to that endured by relatively low standard athletes. Indeed, research (Woodman and Hardy, 1998, 2001; Gould et al., 1999) has suggested that elite performers may be exposed to various kinds of relational and organizational stress before and during major international competitions. Thus, generalized findings with lower-standard sport performers to elite performers might be inappropriate (Hardy et al., 1996; Balague, 1999). Further research with truly high-standard performers is needed to enhance our understanding of the effects of competitive anxiety in an elite sporting environment comparing gender differences. Also, research investigating female athletes in high-standard environments would be particularly helpful, as most studies of high-standard athletes as reported in the meta-analysis by Woodman and Hardy (2003) were men and that sex as a moderator variable may be confounded by the standard of competition. Future investigation involving high-standard women athletes should help to clarify this issue (Cazenave et al., 2007).

The lower cognitive anxiety intensity experienced by the semi-elite (national) male group may be due to individuals’ relative lack of competitive experience and a corresponding lower emotional investment or personal expectations about upcoming task. The participants may be less conversant of their pre-competitive states since this was their first experience reporting their emotional episodes and, may therefore less accurately report their anxiety symptoms (Perry and Williams, 1998).

The multidimensional competitive anxiety predictions were also examined. The current findings further challenges the cognitive anxiety intensity stability during pre-competition whereas somatic anxiety intensity initial stability claim is also questioned although the sudden rise just before competition is supported. A declined in both cognitive and somatic anxiety intensity was observed 2 days prior to competition after an initial increase, 7 days before. This significantly increased 1 h before event depicted a temporal fluctuation during the preparation period. Indeed, current empirical findings are clearly contradictory to the MAT. While in some studies the cognitive subcomponent remained relatively stable across time (Gould et al., 1984; Caruso et al., 1990; Martens et al., 1990), other investigations revealed an increase as the competition neared (Swain and Jones, 1993; Slaughter et al., 1994; Davis and Gill, 1995; Diez and Rosa, 1996; Hall et al., 1998; Cerin et al., 2001). These contradictions in current anxiety literature could be due to the lack of precision in defining the concept of competitive anxiety and the poor construct validity of the CSAI-2 subscales (Lane et al., 1999) as opposed to the inclusion of additional anxiety dimensions (direction and frequency) view (Jones, 1995; Woodman and Hardy, 2001).

Current findings also support the notion about the differentiation of symptom intensity and direction because the directional measure is quite sensitive when distinguishing between person and situational individual difference variables compared to response intensity alone (Jones and Hanton, 2001; Mellalieu et al., 2003). The fact that the elite female athletes perceive their somatic anxiety as more facilitative, while exhibiting appreciable level of self-confidence further supports the “confident coping” notion (Jones and Hanton, 2001). Surprisingly, both international and national male athletes compared to their female counterparts exhibited lower self-confidence levels, which possibly explain their debilitative somatic response. Regardless of the physiological symptoms experienced, performers may be primarily concerned with the cognitive state in a form of worry or apprehension (Jones and Hanton, 2001). The very threat of playing “a relatively good opponent,” the competitiveness of upcoming event, and the thoughts of making the final team could have heightened some physiological reactions which were perceived in a negative manner. This contradicts previous findings that while male athletes generally demonstrate greater confidence than females (Lirgg, 1991; Krane and Williams, 1994), they are also less susceptible to changes in self-confidence during the pre-competition period (Jones and Cale, 1989; Jones et al., 1991). Although speculative, it is possible that continuous perceived pressure, expectations, and distractions of the males’ competition could have rendered elite male athletes confidence atypically “fragile” (Gould et al., 1999). The no gender difference between the elite group in self-confidence may be attributed to the somewhat high level of sport involvement of these athletes and possibly mental skill development acquired over the years through experience from significant others [coaches, experience team mates, friends, and family, (Modroño and Guillen, 2011)]. Future researchers need to consider gender when examining directional anxiety interpretations. If the present findings are replicated, it will then mean that males likewise need more interventions to help them handle anxiety responses more positively.

This empirical investigation has demonstrated that performers with a facilitative interpretation of anxiety symptoms also report greater levels of self-confidence when compared to performers who interpret symptoms as debilitative, especially when the athletes are elite (Jones et al., 1993, 1994; Jones and Swain, 1995; Hanton and Jones, 1997; Perry and Williams, 1998; Hanton et al., 2003). Indeed, self-confidence has been identified as an important variable in the intensity-direction relationship to the extent that it is proposed to potentially “protect” against debilitative interpretations within elite athletes (Hardy et al., 1996). Both qualitative and quantitative studies have offered explanations about the mediating influence of self-confidence when individuals experience anxiety symptoms and their subsequent interpretation toward upcoming performance (Hanton and Connaughton, 2002; Hanton et al., 2004a; Mellalieu et al., 2006b). Self-confidence is suggested to follow an individual’s directional interpretation of anxiety, with increases or decreases in perceived confidence to either improve or lower performance. Thus, high self-confidence level is thought to protect against debilitating interpretations of competitive anxiety in elite athletes.

Contrary to the proposed hypothesis, time-to-competition changes were observed in the direction dimension for cognitive, somatic anxiety and self-confidence with small to large effect size values (0.16, 0.14, and 0.94, respectively). Specifically, interpretation of cognitive anxiety became more positive (or less negative) 1 h before competition whereas somatic anxiety was most debilitative 2 days before competition but slightly improved 1 h before event. These results reinforce similar findings 1 week before competition although both anxiety constructs reported were debilitative (Thomas et al., 2004). The present findings rather conflict with the work of Wiggins (1998), who suggested that perceptions of anxiety did not vary as a function of time-to-competition. The temporal patterns noted for direction in this study support the argument that performers were possibly distinguishing between preparatory and performance anxiety (Burton, 1998) and that the high effect size noted for self-confidence may have acted as a potential buffer toward a more facilitative anxiety interpretation. These findings suggest that sport psychology practitioners and researchers should examine whether interventions to increase self-confidence may result in decreased anxiety symptoms or related anxiety interpretations symptoms as more favorable toward performance (Perry and Williams, 1998).

Due to limited amount of extant literature currently available on the frequency competitive anxiety intrusions due to its relative infant status, arguments put forward for group differences observed in this study would be speculative. Indeed, if somatic anxiety is generally considered as classically conditioned or a reflexive response as a result of environmental stimulus or cues associated with the onset of competition, then the very onset of increases in any physiological arousal prior to competition may be as a result of frequency of competition-related cognitive intrusions reaching some critical or alarming threshold (Jones, 1995; Mellalieu et al., 2004). This may trigger continuous thinking and subsequent increases in worry from the perceived competition environment for elite male performers. These increases in perceived pressure are often accompanied by performance demands associated with competing at a high level (Modroño and Guillen, 2011). The mere thinking of an equally good opponent or opposition at different stages of preparation as competition neared whiles completing the inventory could have triggered appreciable increases in male performers’ somatization. An athlete may mentally be picturing him or herself at the competition site. Subconsciously, many of these athletes may be exposed to the environmental stimuli that are thought to elicit some physiological arousals (Jones et al., 1991).

With large effect size (0.99), present findings showed that frequency of cognitive intrusions increased progressively throughout the pre-competition phase. This is not surprising because table tennis in motor behavior terms involves discontinuous tasks of short duration. Therefore, technical faults with strokes and movement patterns could have triggered intermittent worry and apprehension in between set plays. This possibly explains the somatization responses reported due to perhaps distortions in the neuro-muscular mechanisms. This result replicates the findings noted in similar studies (Swain and Jones, 1993; Hanton et al., 2004b). It is clear from preceding discourse, therefore, that the intensity-directional alone approach which is more prevalent in anxiety literature provides only a limited perspective on the experience of pre-competition anxiety states (Jones, 1995; Edwards and Hardy, 1996; Butt et al., 2003). The notion of frequency of cognitive intrusions provides an avenue for further empirical testing aimed at furthering a detailed understanding of anxiety responses. Practical significance also holds for sport psychologists performing different consultative roles with sport performers at various levels of competition (Jones, 1995).

Recent advances in the competitive anxiety literature have explicitly dichotomized skill level and the notion of competitive experience in sport with respect to the intensity and direction of symptoms associated with anxiety. Although skill level has received considerable research attention, studies on the amount of experience an individual possesses in their respective sport are sparse. Further investigations are required to detail these experiences across other sport types and classifications with and/or without a skill-level criterion, with particular interest on the time scale and amount of competitive experience acquired by performers (Cerin et al., 2000; Mellalieu et al., 2004; Hanton et al., 2007, 2008).

### Limitations

The small effect sizes noted in some areas of the results mean that the practical significance of those findings should be noted with caution. However, we do acknowledge the fact that under certain circumstances, a small effect size would be an important finding. According to Pittenger (2003), when studying a complex social behavior (e.g., anxiety) in a natural environment, one has little direct control over the environment and the treatment of participants. As such measurement procedures may be prone to much error. The inclusion of directional and frequency scales to the CSAI original inventory makes the questionnaire rather arduous to complete, particularly in terms of memory disturbance as competition neared and other social desirability issues inherent in self-reported measures.

## Conclusion

From a theoretical and practical perspective, the current findings require further replication in that the present investigation may have important implications for practitioners regarding the development and implementation of appropriate psychological skills training programs in which design, content and timing should be composite and critical. This would help combat the detrimental consequences of high levels of anxiety during in-season competitive cycle usually associated with professional leagues. To help achieve this, Dias et al. (2012) suggested that athletes’ education and training must also involve coping strategies that promote their cognitive and behavioral flexibility and more adaptive cognitive and behavioral appraisal processes. The successful implementation of ESM contributed in eliciting the dynamic image of athletes’ emotional experiences in this study. According to Cerin et al. (2001), its efficiency rests with participants focus on their momentary experience and in so doing minimizes expectancy effects and memory distortions. Notably, it allows the analysis of relationships between transient situational variables and cognitive contents as well as other athletes’ emotional experience. Therefore, the implementation of this method may greatly contribute to a better understanding of the complex cognitive and emotional reactions of athletes in the period leading up to a major athletic event.

## Author Contributions

All authors substantially contributed to the conception or design of the work; or the acquisition, analyses, and interpretation of data, drafted the initial manuscript and its final approval.

## Conflict of Interest Statement

The authors declare that the research was conducted in the absence of any commercial or financial relationships that could be construed as a potential conflict of interest.
